# Increased Social Support Reduces the Incidence of Motoric Cognitive Risk Syndrome

**DOI:** 10.1093/geroni/igac048

**Published:** 2022-08-08

**Authors:** Nicole Felix, Emmeline Ayers, Joe Verghese, Helena M Blumen

**Affiliations:** Department of Medicine, Albert Einstein College of Medicine, Bronx, New York, USA; Departments of Neurology, Albert Einstein College of Medicine, Bronx, New York, USA; Department of Medicine, Albert Einstein College of Medicine, Bronx, New York, USA; Departments of Neurology, Albert Einstein College of Medicine, Bronx, New York, USA; Department of Medicine, Albert Einstein College of Medicine, Bronx, New York, USA; Departments of Neurology, Albert Einstein College of Medicine, Bronx, New York, USA

**Keywords:** Predementia, Preclinical dementia, Slow gait, Subjective cognitive complaint

## Abstract

**Background and Objectives:**

The motoric cognitive risk syndrome (MCR) is a predementia syndrome characterized by slow gait and cognitive complaint. The relationship between MCR and social support—a potentially modifiable risk factor of dementia—is currently unknown. The current study aimed to determine whether MCR incidence varies as a function of social support in aging.

**Research Design and Methods:**

We examined MCR incidence in 506 community-dwelling older adults (*M* Age 76.59; 57.3% female) without MCR or dementia at baseline. We quantified perceived levels of social support with the Medical Outcomes Study Social Support Survey, incorporating four different categories of support: (a) emotional/informational support, (b) tangible support, (c) affectionate support, and (d) positive social interactions. We used Cox regression analyses, adjusted for age, sex, race/ethnicity, education, marital status, comorbidities, and global cognition, to estimate hazard ratios (aHR) with 95% confidence intervals (CIs).

**Results:**

Over a median follow-up time of 2.5 years (range = 1–7 years), 38 participants (9.8%) developed MCR. Increased tangible support decreased the risk of MCR by 30% (aHR: 0.70, 95% CI: 0.53–0.92, *p* = .011). Increased overall social support decreased the risk of MCR by 33% (aHR: 0.67, 95% CI: 0.46–0.98, *p* = .038). Other subcategories of social support were not associated with a decreased risk of MCR (*p* > .05).

**Discussion and Implications:**

Higher levels of tangible social support, as well as overall social support, were associated with reduced risk for MCR in older adults. Increasing social support may be a promising avenue of intervention for reducing the risk of MCR, dementia, and other forms of cognitive decline.

**Translational Significance:** This study examined the relationship between perceived social support and the motoric cognitive risk syndrome (MCR)—a predementia syndrome that is characterized by slow gait and subjective cognitive complaint. We found that higher levels of social support were associated with a reduced risk for MCR. We conclude that social support is a potentially modifiable risk factor for MCR. There is some evidence that social interventions can improve the well-being of individuals with dementia. Our study highlights the role of social support in reducing the risk of developing MCR and dementia.

The motoric cognitive risk syndrome (MCR) is a predementia syndrome characterized by the presence of slow gait and cognitive complaint in the absence of dementia and mobility disability (Verghese et al., 2012). MCR affects almost 10% of community-dwelling older adults worldwide and consistently predicts cognitive impairment Alzheimer’s disease and vascular dementia (Verghese, Annweiler, et al., 2014; Verghese, Ayers, et al., 2014; Verghese et al., 2012, 2019). In a multicenter study of more than 4,000 older adults, for example, MCR doubled the risk for developing incident cognitive impairment (adjusted hazard ratio [aHR] 2.0, 95% CI: 1.7–2.4) and almost doubled the risk for developing dementia (aHR 1.9, 95% CI: 1.5–2.3; Verghese, Annweiler, et al., 2014).

To date, the risk for MCR has been shown to increase with increasing age and in the presence of several medical conditions and adverse outcomes—including obesity, stroke, hypertension, diabetes, depression, Parkinson disease, and falls (Beauchet et al., 2018; Callisaya et al., 2016; Doi et al., 2015; Verghese, Annweiler, et al., 2014; Verghese, Ayers, et al., 2014). Identifying modifiable risk factors for MCR is important because it can provide insights into the development of prevention and treatment strategies for MCR, cognitive impairment, and dementia. The current study aimed to determine whether MCR incidence varies as a function of social support—a potentially modifiable risk factor for cognitive decline and dementia (Barnes et al., 2004; Bassuk et al., 1999; Khondoker et al., 2017; Seeman et al., 2001).

Social support is the perceived or actual level of assistance or sense of companionship provided by the people in one’s social network (Antonucci, 1990). There is increasing interest in exploring the therapeutic effects of social interventions and increasing government funding for social support, particularly in response to the COVID-19 pandemic. Yet, there is still insufficient data in the literature regarding the preventative effects of social support interventions on MCR, and other forms of cognitive decline. In fact, the efficacy of social prescribing—which aims to connect individuals with non-medical sources of support within their communities—in general remains unclear (Bickerdike et al., 2017; Brown et al., 2021). A recent study, however, suggests that social prescribing improves the mental well-being of individuals with dementia, and their caregivers (Giebel et al., 2021).

We hypothesized that social support is a potentially modifiable risk factor for MCR because poor social support (like MCR) has been linked to cognitive function, cognitive decline, and dementia (Barnes et al., 2004; Bassuk et al., 1999; Khondoker et al., 2017; Seeman et al., 2001). In one study of cognitively healthy older adults, for instance, baseline emotional support was associated with cognitive performance at cross-section, and after 7.5 years. (Seeman et al., 2001). In another study, positive social support from children was associated with a 17% reduced risk for dementia (Khondoker et al. 2017). There is also some evidence that social support, like MCR, is associated with overlapping patterns of brain regions—that are composed of regions that are important for social, cognitive, and motor functions—including prefrontal and insular brain regions (Blumen et al., 2018, 2021; Cotton et al., 2019). A social network encompasses the different kinds of relationships an individual has access to as well as the total number of people an individual interacts with regularly (Antonucci, 1990; Cohen et al., 1997). Social networks have also been linked with both cognitive function, cognitive decline and dementia (Barnes et al., 2004; Bassuk et al., 1999; Ertel et al., 2008; Fratiglioni et al., 2004), and with overlapping patterns of brain regions as MCR (Blumen & Verghese, 2019; Pillemer et al., 2016), and may therefore be another potentially modifiable risk factor of MCR.

The primary aim of this study was to examine MCR incidence as a function of perceived levels of social support among older adults without dementia. Perceived social support was assessed with the Medical Outcomes Study Social Support Survey (MOS-SSS; Sherbourne & Stewart, 1991) because it measures overall support and four different kinds of support: (a) emotional or informational support, (b) tangible support, (c) affectionate support, and (d) positive social interactions. A previous study, which used the MOS-SSS, further suggests that overall social support, emotional/informational support, and positive social interactions are associated with higher global cognition in older adults at cross-section (Pillemer & Holtzer, 2016). Overall support, tangible support, affectionate support, and positive social interactions have also been linked to a decrease in incident cognitive impairment among older men (Pillemer et al., 2019). In addition, we have previously linked overall support and tangible support to gray matter volume, primarily in prefrontal, hippocampal, cingulate, insular, and thalamic brain regions (Cotton et al., 2019), which overlap with brain areas linked to the MCR syndrome (Blumen et al., 2018; 2021). A recent study has also shown that increased social support utilization (or support seeking behaviors) is associated with a reduced risk for MCR in middle-aged to older adults (Sun et al., 2022). Based on these studies—highlighting the shared cognitive outcomes and shared neural substrates of MCR and different kinds of social support, along with initial evidence that support seeking behaviors are associated with a reduced risk for MCR—we hypothesized that increased levels of social support would decrease the risk for developing MCR.

The secondary aim of this study was to examine MCR incidence as a function of social network size and social network diversity among older adults without dementia. Social networks were assessed with the social network index (SNI; Cohen et al., 1997), which provides two important measures: a measure of the number of high-contact social roles (spouse, parent, child, child-in-law, close relative, close friend, religious group member, student, employee, neighbor, volunteer, and group member) as a means to evaluate social network diversity, as well as the total number of high-contact social roles a person interacts with at least biweekly in order to evaluate total network size. Increased social network diversity, not just social network size, has been associated with higher global cognitive function (Ali et al., 2018). Like the MOS-SSS, the SNI is associated with gray matter volume in prefrontal, hippocampal, cingulate, insular, and thalamic brain regions (Blumen & Verghese et al., 2019). The SNI is also similar to another social network measure that quantifies the number of social relationships that a person interacts with at least monthly, which has been shown to predict cognitive decline in older African Americans and Whites (Barnes et al., 2004)—and to mediate the relationship between autopsy-confirmed Alzheimer’s disease pathology and cognitive function in older adults (Bennett et al., 2006). Thus, based on these studies underscoring the shared cognitive outcomes and the shared neural substrates of MCR and social networks, we hypothesized that participants with a higher number of high-contact social roles and larger social networks would have a decreased risk of developing MCR.

## Method

### Participants

This prospective cohort study examined MCR incidence in a sample of 506 adults aged 65 and older enrolled in the Central Control of Mobility in Aging (CCMA) study (Holtzer et al., 2014). The goal of the CCMA study, which took place in 2011–2018, was to identify cognitive and brain predictors of mobility. Older adults (>65 years) residing in Yonkers and Westchester (NY) were first contacted via mail and then over the phone. Over the phone, they provided verbal consent and completed a brief medical history questionnaire, a life space assessment (Harada et al., 2010), the AD8 (an 8-item informant interview to detect dementia; Galvin et al., 2005), and the Memory Impairment Screen (Buschke et al., 1999; Lipton et al., 2003). General exclusion criteria included severe auditory or visual loss, recent hospitalization that affects mobility, living in a nursing home, serious chronic or acute illness (e.g., cancer), and the presence of dementia or other neurodegenerative disease. Participants who were eligible for this analysis did not meet criteria for either MCR or dementia at their baseline evaluation, and MCR criteria was evaluated annually (at each wave). Social support and social networks were also assessed annually, but only baseline scores were utilized in the analysis because our study was not looking at how these components changed over time—but whether baseline scores predicted incidence MCR. The study protocol was approved by the Einstein institutional review board. All participants signed informed consent prior to enrollment.

### Predictor, Outcome, and Covariates

#### Perceived social support

Perceived social support was assessed with the Medical Outcomes Study Social Support Survey (MOS-SSS; Sherbourne & Stewart, 1991) by research assistants at each study visit because it quantifies perceived social support in four previously validated categories (Gjesfjeld et al., 2008): (a) emotional or informational support (e.g., someone who understands your problems), (b) tangible support (e.g., someone to help you if you are confined to bed), (c) affectionate support (e.g., someone who shows you love and affection), and (d) positive social interactions (e.g., someone to have a good time with). Participants were asked to report to what extent they agreed with each statement on a Likert scale including the following choices: none of the time, a little of the time, some of the time, most of the time, and all of the time. Adding these four subcategories of social support together can also be used to measure a person’s overall level of social support. Emotional support reflects the expression of positive affect, empathetic understanding, and the encouragement of expressions of feelings; informational support is characterized as the receipt of advice, information, guidance and/or feedback; tangible support is the provision of behavioral and physical assistance; affectionate support includes expressions of love or affection; and positive social interactions reflects the availability of someone to carry out fun activities with.

#### Social networks

We utilized the Social Network Index (SNI; Cohen et al., 1997) to determine social network diversity evaluated by the number of high-contact relationship types (SNI-1) and the total network size (SNI-2) for each participant. The SNI-1 quantifies biweekly interactions with 12 different types of social relationships domains: spouse, parents, parents-in-law, children, children-in-law, other close family members, neighbors, work colleagues, school peers, fellow volunteers, members of religious groups, and members of nonreligious groups. The SNI-2 quantifies the total number of people an individual interacts with at least biweekly across these domains to capture total network size.

#### Motoric cognitive risk syndrome

MCR was defined as slow gait and subjective cognitive complaint among older adults without dementia and preserved activities of daily living. The MCR criteria applied at each wave were blinded to any previous diagnosis or information. Older adults diagnosed with dementia using the fourth edition of the *Diagnostic and Statistical Manual of Mental Disorders* (DSM-IV; American Psychological Association, 1994) at consensus case conferences attended by a neurologist and neuropsychologist with access to all clinical and neuropsychological information procedure were excluded at baseline as well as if they had met criteria on follow-up (if they did not meet MCR criteria at a prior visit). Gait speed (cm/s) at normal pace was quantified on a 20 foot (609.6 cm) instrumented walkway (GaitRite System, Clifton, NJ). Slow gait was defined as gait speed one standard deviation or more below age- and sex-specific means based on previously established diagnostic procedures (Verghese, Annweiler, et al., 2014; Verghese, Ayers, et al., 2014). The average gait speed in this study was 100.6 cm/s. Subjective cognitive complaint was obtained from positive responses on the memory item on the Geriatric Depression Scale (GDS), which asks participants the following yes or no question: “Do you feel you have more problems with memory than most?” (GDS; Yesavage et al., 1982) or a score of 1 or more on the AD8 Questionnaire (Galvin et al., 2005).

#### Covariates

Covariates were selected based on previously reported associations with MCR, social support, and/or cognitive decline. A comorbidity score (range 0–10) was obtained from self-reported presence of physician diagnosed diabetes, chronic heart failure, arthritis, hypertension, depression, stroke, chronic obstructive pulmonary disease, angina, Parkinson’s disease, and myocardial infarction. A dichotomized variable of sex/gender (male/female) was obtained from self-report. Marital status was categorized into four categories (a) currently married and living together, (b) never married and never lived with someone in a marital-like relationship, (c) separated/divorced/formerly lived with someone in a marital-like relationship, or (d) widowed. Race/ethnicity was ascertained by self-report and was categorized as (a) White, (b) Black, (c) Hispanic, (d) Asian, (f) Other, or (g) missing values. Education was obtained by self-reported years of education. The GDS 30-item scale was used to quantify depressive symptoms. Lastly, a global cognition score was represented by the Repeatable Battery for the Assessment of Neuropsychological Status (RBANS) total score (Duff et al., 2008; Randolph et al., 1998).

### Statistical Approach

Cox proportional hazard models adjusted for age, sex, race/ethnicity, marital status, years of education, and comorbidity score were used to estimate hazard ratios (aHR) with 95% confidence intervals (CIs) for developing incident MCR as a function of perceived levels of social support and social networks at baseline. Separate models were run to examine the association with each exposure including (a) emotional/informational support; (b) tangible support; (c) affectionate support; (d) perceived social interaction; (e) total overall support; (f) SNI-1; and (g) SNI-2. Time to event was measured in years from baseline to the first visit at which MCR was diagnosed or to final study contact, whichever came first. The median follow-up time was 2.5 years (range = 1–7 years). We excluded any participant diagnosed with MCR or dementia at baseline. Incident dementia was not excluded since we expected some individuals with MCR to develop dementia during follow-up. Proportional hazards assumptions were tested statistically and graphically for all our variables and covariates and were adequately met for all analyses. All analyses were conducted using SPSS Statistics version 24.

We conducted multiple sensitivity analyses. First, in addition to dementia and MCR, we excluded any participants with baseline and incident mild cognitive impairment (MCI; Petersen et al., 1999, 2009) and reran our Cox models. MCI status was determined using the Petersen et al., (1999) criteria, at the consensus diagnostic conferences described above. Second, depression has been reported to predict incident MCR (Verghese, Ayers, et al., 2014). Therefore, we ran a follow-up analysis where we excluded history of depression from the comorbidity score and added depressive symptoms (GDS score) as a covariate. To ensure that we did not overadjust by including the global cognition score as a covariate, we also ran all the models without adjusting for global cognition (RBANS total).

## Results

Data from a total of 506 CCMA participants with up to 7 years of follow-up (median 2.5 years; see Table 1) were included, after excluding individuals with MCR (*n* = 39) or dementia (*n* = 9) at baseline. Thirty-eight participants (7.5%) developed MCR, and the average time to diagnosis was 2.56 years. Bivariate statistics determined that individuals who developed MCR were significantly older, had more comorbidities, higher GDS scores, and were less likely to be married, compared with those who did not develop MCR. Groups did not significantly differ in terms of sex, gender, race/ethnicity, RBANS total, or years of education. These results were somewhat consistent with previous literature (Doi, et al., 2015; Verghese, Annweiler, et al., 2014) in that age and medical conditions were associated with an increased risk in MCR. However, in our study, years of education and other covariates were not significantly associated with MCR. Note that participants without follow-up measures (*N* = 117) automatically do not contribute to the results of Cox proportional hazard models. Those without follow-up data did not differ from those with follow-up data in terms of age, years of education, sex, GDS scores, gender, race/ethnicity, or marital status, but they had significantly lower RBANS total scores, affectionate support, positive social interactions, and overall support (see Supplementary Table 1).

**Table 1.** Baseline Characteristics of Individuals That Developed and Did Not Develop MCR During Follow-up

Characteristic	No MCR (*n* = 468)		MCR (*n* = 38)	
	Mean ± *SD*	*n* (%)	Mean ± *SD*	*n* (%)
Age	76.30 ± 6.35		79.79 ± 6.56	
Years of education	14.69 ± 2.93		14.66 ± 2.72	
Comorbidity score (range = 0–10)^a^	1.60 ± 1.08		2.00 ± 0.90	
Geriatric Depression Scale (range = 0–30)	4.27 ± 3.62		6.76 ± 4.54	
Female		260 (55.6)		21 (55.3)
Global cognition (RBANS total)	92.12 ± 11.87		91.68 ± 8.98	
Race/ethnicity				
Caucasian		377 (80.6)		31 (81.6)
Black		71 (15.2)		7 (18.4)
Hispanic		11 (2.3)		—
Asian		6 (1.3)		—
Other		3 (0.6)		—
Marital status				
Married		196 (42.8)		13 (34.2)
Never married		33 (7.2)		1 (2.6)
Divorced/separated		80 (17.5)		3 (7.9)
Widowed		149 (32.5)		21 (55.3)
MOS-SSS				
Emotional/informational support	4.06 ± .95		3.73 ± 1.07	
Tangible support	4.01 ± 1.13		3.63 ± 1.46	
Affectionate support	4.34 ± .90		4.14 ± 1.01	
Positive social interactions	4.17 ± .94		3.93 ± 1.09	
Total overall support	4.11 ± .82		3.80 ± 0.95	
SNI				
SNI-1 (social network diversity)	5.25 ± 1.55		4.95 ± 1.45	
SNI-2 (social network size)	27.85 ± 44.11		19.71 ± 11.95	

*Notes:* MCR = motoric cognitive risk syndrome; RBANS = Repeatable Battery for the Assessment of Neuropsychological Status; MOS-SSS = Medical Outcomes Study Social Support Survey; SNI = Social Network Index.

^a^Comorbidity score was obtained from dichotomous ratings of diabetes, chronic heart failure, arthritis, hypertension, depression, stroke, Parkinson’s diseases, chronic obstructive pulmonary disease, angina, and myocardial infarctions.

### Social Support

Of the four categories of social support, only increased tangible support decreased the risk of MCR by 30% (aHR: 0.70, 95% CI [0.53–0.92], *p* = .011), even after adjusting for age, sex, race/ethnicity, marital status, years of education, comorbidity score, global cognition score, and geriatric depression scale. A higher overall social support score additionally decreased the risk of MCR by 33% (aHR: 0.67, 95% CI [0.46–0.98], *p* = .038), whereas the other categories of social support were not significantly associated with a decreased risk of MCR (*p* > .05; see Table 2).

**Table 2.** Association of Social Support and Social Network Index Scores With Incident MCR, After Excluding Individuals With MCR and Dementia at Baseline. Adjusted for Age, Sex, Education, Race/Ethnicity, Marital Status, Global Cognition (RBANS Total), and Comorbidity Score

Variable	Adjusted HR (95% CI)	*p*-Value
Emotional/informational support	0.750 (0.541–1.040)	.850
Tangible support	0.696 (0.526–.922)	.011*
Affectionate support	0.752 (0.517–1.095)	.137
Positive social interactions	0.848 (0.611–1.178)	.325
Total overall support	0.670 (0.460–.977)	.038*
SNI-1 (social network diversity)	0.855 (0.678–1.079)	.187
SNI-2 (total network size)	0.978 (0.950–1.007)	.133

*Notes:* CI = confidence interval; HR = harzard ratio; MCR = motoric cognitive risk syndrome; RBANS = Repeatable Battery for the Assessment of Neuropsychological Status; SNI = Social Network Index.

*p < .05

### Social Network Index

Social network size and social network diversity were not associated with incident MCR in our main analyses (Table 2), or the sensitivity analyses discussed below (Supplementary Tables 2–6).

### Sensitivity Analyses

First, we calculated risk of MCR after excluding participants with MCI at baseline. Excluding individuals with MCI at baseline (*n* = 60) left us with 442 cases of the original 506. Thirty-three participants (7.5%) developed MCR. Once again, tangible support had a significant protective effect against MCR by 30.7% (aHR: 0.693, 95% CI [0.511–.938], *p* = .018), but the overall social support score was only marginally significant (*p* = .074; Supplementary Table 2). When we removed depression from our comorbidity score and added the GDS score as a covariate, tangible support remained significantly protective against MCR (Supplementary Table 2). This result was consistent whether we included individuals with MCI at baseline (aHR: 0.713, 95% CI [0.531–0.957], *p* = .024; Supplementary Table 3) or excluded them (aHR: 0.700, 95% CI [0.511–0.957], *p* = .026; Supplementary Table 2)—and when we excluded individuals with incident MCI (aHR: 0.591, 95% CI [0.412–0.847], *p* = .004; Supplementary Table 4). The overall social support score, however, was no longer significant in these models. When we excluded 14 participants who developed MCR within the first year of follow-up, additional measures of social support became significant in certain models, but only tangible support remained significantly protective against incident MCR in all models (Supplementary Tables 5 and 6). Finally, when we ran all models without adjusting for global cognition, similar patterns of results were observed. For example, the equivalent model to the one ran in Table 2 without global cognition as a covariate resulted in tangible support (aHR: 0.69, 95% CI: 0.52–0.92, *p* = .010) and overall social support (aHR: 0.66, 95% CI: 0.47–0.92, *p* = .013) being protective against the development of MCR.

## Discussion

The key findings of this study were that high levels of perceived social support—particularly tangible and overall support—were associated with a reduced risk for developing MCR, a preclinical stage of dementia characterized by slow gait and cognitive complaint. These results were observed even after adjusting for age, sex, race/ethnicity, years of education, comorbidity, depressive symptoms, MCI (baseline and incident), and marital status, as well as in sensitivity analyses that excluded participants who developed MCR within the first year. Tangible support in this study was characterized as direct physical assistance including having someone to help you (a) if you were confined to a bed, (b) take you to the doctor if you needed it, (c) prepare your meals if you were unable to do it yourself, and (d) help you with daily chores if you were sick. Overall support was the sum of tangible support, emotional/informational support, affectionate support, and positive social interactions. The number of high-contact social roles and total network size, however, was not associated with MCR incidence in this study.

These results are consistent with previous studies that have linked social support to cognitive decline, MCI, and dementia (Barnes et al., 2004; dos Santos et al., 2018; Khondoker et al., 2017; Pais et al., 2021; Seeman et al., 2001)—but also extend them to show that robust social support reduces the risk for MCR, independent of MCI and dementia, and after adjusting for a number of potential confounders, including depressive symptoms. These findings highlight the benefit of evaluating different kinds of social support in identifying individuals at increased risk for MCR. The modest (~40%) overlap between MCR and MCI observed in previous large-scale and multicountry studies (Verghese, Annweiler, et al., 2014; Verghese, Ayers, et al., 2014) further suggests that MCR likely captures a different or larger group of older adults at increased risk for dementia. The clinical utility of MCR is also notable because it can be quickly diagnosed in most clinical and research settings without specialized equipment or personnel.

These findings also support investments in therapeutic strategies to increase social support, particularly tangible support, for reducing the risk of MCR, dementia, and other forms of cognitive decline. It is also important to acknowledge that in our study, the association between overall social support and MCR may be primarily driven by tangible support. Interestingly, we previously found that overall support and tangible support (but not the other categories of support assessed with the MOS-SSS) were linked to a pattern of gray matter volume primarily composed of prefrontal, hippocampal, insular, cingulate, and thalamic brain regions (Cotton et al., 2019). More specifically, increased overall and tangible support was associated with greater gray matter volume in these brain regions. We have also found that some of these brain regions (prefrontal and insular brain regions) are particularly affected by MCR (Blumen et al., 2018). More specifically, older adults with MCR had less gray matter volume in these brain regions than older adults without MCR. Thus, social support may reduce the risk for MCR because it engages or strengthens the neural substrates that contribute to MCR. Future studies, however, are needed to identify specific methods for providing social support—specifically tangible support—and determine their potential for strengthening these neural substrates, and reducing the risk for MCR, dementia, and other forms of cognitive decline.

There are both strengths and limitations to this study. Notable strengths of this study include (a) the use of comprehensive and previously validated scales of perceived social support and social networks, (b) large sample size (*N* = 506) and long annual follow-up (up to 7 years) that enabled to account for confounders and bias, (c) careful screening of individuals for dementia and MCI, (d) carefully developed hypotheses in the context of existing literature, and (e) thorough consideration of potential confounders such as depressive symptoms. Despite carefully designed hypotheses and consideration of potential confounders, we chose not to adjust for multiple comparisons—and therefore, these findings need to be confirmed in future studies. The generalizability of this study is also limited by that the older adults in our sample were largely white, highly educated, and with relatively few comorbidities. Future studies that directly address these issues in racially and ethnically diverse samples of older adults with more varied levels of education and physical health are needed. Future studies are also needed to determine whether the relationship between social support and MCR incidence is influenced by other protective factors for MCR, including personality traits (Ayers et al., 2020; Stephan et al., 2020).

Social prescribing is already utilized to address healthcare needs, even if additional studies are needed to establish efficacy (Bickerdike et al., 2017; Brown et al., 2021). Our study highlights the potential for social support, particularly tangible support, to prevent the development of MCR, cognitive decline, cognitive impairment, and dementia. Studies that build upon current social prescribing methods in order to increase social support should be developed and systematically examined in older adult populations. The potential benefits of increasing tangible support in older adult populations could also be studied within the context of home health services provided to assist older adults with daily activities. Overall, this study suggests that social support is a potentially modifiable risk factor for MCR and encourages the development of interventions that strengthens social support as effective means of prevention of MCR.

## Supplementary Material

igac048_suppl_Supplementary_Material

## Data Availability

This study was not preregistered. The data used in this article can be made available upon request.
