# Supplementary material for: Increased Social Support Reduces the Incidence of Motoric Cognitive Risk Syndrome
Source: Innov Aging. 2022 Aug 8;6(5):igac048. doi: 10.1093/geroni/igac048 (PMC9447853; doi:10.1093/geroni/igac048)
Supplement: igac048_suppl_Supplementary_Material [file igac048_suppl_supplementary_material.docx]

**Supplementary Table 1.** Comparison of those with and without follow-up data.

| **Characteristic** | **Follow-up (*n*=389)** | | **No follow-up (*n*=117)** | | **p value** |
| --- | --- | --- | --- | --- | --- |
|  | **Mean ± SD** | ***n* (%)** | **Mean ± SD** | ***n* (%)** |  |
| Age | 76.63 ± 6.33 |  | 76.31 ± 6.74 |  | 0.634 |
| Years of Education | 14.77 ± 2.87 |  | 14.44 ± 3.03 |  | 0.295 |
| Comorbidity Score (range = 1-10) ^a^ | 1.63 ± 1.05 |  | 1.61 ± 1.16 |  | 0.897 |
| Geriatric Depression Scale (range = 0-30) | 4.28 ± 3.57 |  | 5.03 ± 4.26 |  | 0.147 |
| Female, n (%) |  | 222 (57.1) |  | 59 (50.4) | 0.205 |
| Global Cognition (RBANS total**) | 92.89 ± 11.70 |  | 89.42 ± 11.19 |  | 0.005 |
| Race/Ethnicity |  |  |  |  | 0.425 |
| Caucasian |  | 316 (81.2) |  | 92 (78.6) |  |
| Black |  | 59 (15.2) |  | 19 (16.2) |  |
| Hispanic |  | 6 (1.5) |  | 5 (4.3) |  |
| Asian |  | 5 (1.3) |  | 1 (0.9) |  |
| Other |  | 3 (0.8) |  | -- |  |
| Marital Status |  |  |  |  | 0.809 |
| Married |  | 164 (42.3) |  | 45 (41.7) |  |
| Never Married |  | 26 (6.7) |  | 8 (7.4) |  |
| Divorced/Separated |  | 62 (16.0) |  | 21 (19.4) |  |
| Widowed |  | 136 (35.1) |  | 34 (31.5) |  |
| MOS-SSS |  |  |  |  |  |
| Emotional/Informational support | 4.06 ± 0.94 |  | 3.95 ± 1.04 |  | 0.318 |
| Tangible support | 4.04 ± 1.14 |  | 3.80 ± 1.23 |  | 0.055 |
| Affectionate support | 4.39 ± 0.84 |  | 4.11 ± 1.09 |  | 0.030 |
| Positive Social Interactions | 4.22 ± 0.89 |  | 3.93 ± 1.11 |  | 0.017 |
| Total Overall Support | 4.13 ± 0.81 |  | 3.93 ± 0.91 |  | 0.036 |
| SNI |  |  |  |  |  |
| SNI-1 | 5.28 ± 1.49 |  | 5.04 ± 1.73 |  | 0.102 |
| SNI-2 | 27.73 ± 46.14 |  | 25.41 ± 26.03 |  | 0.970 |

*Notes.*  MCR = motoric cognitive risk syndrome; RBANS = Repeatable Battery for the Assessment of Neuropsychological Status; MOS-SSS = Medical Outcomes Study Social Support Survey; SNI = Social Network Index.

^a^ Comorbidity score was obtained from dichotomous ratings of diabetes, chronic heart failure, arthritis, hypertension, depression, stroke, Parkinson’s diseases, chronic obstructive pulmonary disease, angina, myocardial infarctions.

**Supplementary Table 2.** Association of Social Support and Social Network Index scores with incident MCR, after excluding individuals with MCR, Dementia and MCI at baseline. Adjusted for age, sex, education, race/ethnicity, marital status, global cognition (RBANS total) and comorbidity score.

| **Variable** | **Not adjusted for GDS Score** | | **Additionally Adjusted for GDS** | |
| --- | --- | --- | --- | --- |
|  | **Adjusted HR (95% CI)** | **P value** | **Adjusted HR (95% CI)** | **P value** |
| Emotional/Informational Support | 0.776 (.533-1.130) | 0.186 | 0.870 (.583-1.299) | 0.496 |
| Tangible Support | 0.693 (.511-.938) | 0.018* | 0.700 (.511-.957) | 0.026* |
| Affectionate Support | 0.778 (.517-1.168) | 0.226 | 0.847 (.551-1.300) | 0.447 |
| Positive Social Interactions | 0.837 (.590-1.187) | 0.318 | 0.959 (.665-1.382) | 0.821 |
| Total Overall Support | 0.683 (.450-1.038**)** | 0.074 | 0.763 (.491-1.188) | 0.231 |
| SNI-1 (social network diversity) | 0.899 (.700-1.154) | 0.403 | .941 (.716-1.237) | 0.664 |
| SNI-2 (total network size) | .986 (.958-1.015) | 0.344 | .992 (.964-1.021) | 0.583 |

*Notes.* MCR = motoric cognitive risk syndrome; MCI = mild cognitive impairment; RBANS = Repeatable Battery for the Assessment of Neuropsychological Status; GDS = Geriatric Depression Scale; SNI = Social Network Index.

**Supplementary Table 3.** Association of Social Support and Social Network Index scores with incident MCR, after excluding individuals with MCR and Dementia. Adjusted for age, sex, education, race/ethnicity, marital status, global cognition (RBANS total) comorbidity score, as well as depressive symptoms (GDS score).

| **Variable** | **Adjusted HR (95% CI)** | **P value** |
| --- | --- | --- |
| Emotional/Informational Support | 0.826 (.585-1.167) | 0.278 |
| Tangible Support | 0.713 (.531-.957) | 0.024* |
| Affectionate Support | 0.825 (.559-1.219) | 0.335 |
| Positive Social Interactions | 0.959 (.679-1.356) | 0.813 |
| Total Overall Support | 0.743 (.498-1.108) | 0.145 |
| SNI-1 (social network diversity) | .918 (.716-1.178) | 0.503 |
| SNI-2 (total network size) | .984 (.955-1.014) | 0.292 |

*Notes.* MCR = motoric cognitive risk syndrome; RBANS = Repeatable Battery for the Assessment of Neuropsychological Status; GDS = Geriatric Depression Scale; SNI = Social Network Index.

**Supplementary Table 4**. Association of Social Support and Social Network Index scores with incident MCR, after excluding individuals with MCR, dementia and MCI, and those who developed MCI prior to MCR. Adjusted for age, sex, education, race/ethnicity, marital status, global cognition (RBANS total) comorbidity score, as well as depressive symptoms (GDS score).

| **Variable** | **Adjusted HR (95% CI)** | **P value** |
| --- | --- | --- |
| Emotional/Informational Support | 0.879 (.554-1.393) | 0.582 |
| Tangible Support | 0.591 (.412-.847) | 0.004* |
| Affectionate Support | 0.790 (.485-1.286) | 0.343 |
| Positive Social Interactions | 0.778 (.522-1.160) | 0.218 |
| Total Overall Support | 0.681 (.419-1.105) | 0.120 |
| SNI-1(social network diversity) | .941 (.716-1.237) | 0.664 |
| SNI-2 (total network size) | .992 (.964-1.021) | 0.583 |

*Notes.* MCR = motoric cognitive risk syndrome; MCI = mild cognitive impairment; RBANS = Repeatable Battery for the Assessment of Neuropsychological Status; GDS = Geriatric Depression Scale; SNI = Social Network Index.

**Supplementary Table 5.** Association of Social Support and Social Network Index scores with incident MCR, after excluding individual with MCR, dementia, and those who developed MCR during the first year of follow-up. Adjusted for age, sex, education, race/ethnicity, marital status, global cognition (RBANS total) and comorbidity score.

| **Variable** | **Not adjusted for GDS** | | **Additionally Adjusted for GDS** | |
| --- | --- | --- | --- | --- |
|  | **Adjusted HR (95% CI)** | **P-value** | **Adjusted HR (95% CI)** | **P-value** |
| Emotional/Informational Support | 0.620 (.415-.927) | 0.020* | 0.688 (.449-1.055) | 0.086 |
| Tangible Support | 0.582 (.405-.834) | 0.003* | 0.597 (.407-.873) | 0.008* |
| Affectionate Support | 0.518 (.331-.813) | 0.004* | 0.573 (.362-.907) | 0.018* |
| Positive Social Interactions | 0.776 (.521-1.155) | 0.211 | 0.862 (.563-1.319) | 0.493 |
| Total Overall Support | 0.519 (.326-.826) | 0.006* | 0.574 (.351-.938) | 0.027* |
| SNI-1 (social network diversity) | .934 (.694-1.257) | 0.653 | 1.006 (.729-1.387) | 0.973 |
| SNI-2 (total network size) | .997 (.975-1.019) | 0.773 | .999 (.981-1.017) | 0.91 |

*Notes.* MCR = motoric cognitive risk syndrome; RBANS = Repeatable Battery for the Assessment of Neuropsychological Status; GDS = Geriatric Depression Scale; SNI = Social Network Index.

**Supplementary Table 6.** Association of Social Support and Social Network Index scores with incident MCR, after excluding individual with MCR, dementia, MCI, and those who developed MCR during the first year of follow-up. Adjusted for age, sex, education, race/ethnicity, marital status, global cognition (RBANS total) and comorbidity score.

| **Variable** | **Not adjusted for GDS** | | **Additionally Adjusted for GDS** | |
| --- | --- | --- | --- | --- |
|  | **Adjusted HR (95% CI)** | **P value** | **Adjusted HR (95% CI)** | **P value** |
| Emotional/Informational Support | 0.584 (.361-.944) | 0.028* | 0.669 (.398-1.122) | 0.128 |
| Tangible Support | 0.579 (.385-.870) | 0.009* | 0.615 (.406-.933) | 0.022* |
| Affectionate Support | 0.535 (.322-.887) | 0.015* | 0.615 (.368-1.028) | 0.064 |
| Positive Social Interactions | 0.765 (.493-1.187) | 0.232 | 0.881 (.552-1.405) | 0.594 |
| Total Overall Support | 0.500 (.293-.855) | 0.011* | 0.582 (.331-1.023) | 0.06 |
| SNI-1 (social network diversity) | .960 (.689-1.336) | 0.807 | .992 (.686-1.434) | 0.965 |
| SNI-2 (total network size) | 1.002 (.985-1.018) | 0.843 | 1.003 (.987-1.019) | 0.709 |

*Notes.* MCR = motoric cognitive risk syndrome; MCI = mild cognitive impairment; RBANS = Repeatable Battery for the Assessment of Neuropsychological Status; GDS = Geriatric Depression Scale; SNI = Social Network Index.
